# Surveillance of a 2D Plane Area with 3D Deployed Cameras

**DOI:** 10.3390/s140201988

**Published:** 2014-01-24

**Authors:** Yi-Ge Fu, Jie Zhou, Lei Deng

**Affiliations:** Department of Automation, Tsinghua University, Beijing 100084, China; E-Mails: jzhou@tsinghua.edu.cn (J.Z.); dengl09@mails.tsinghua.edu.cn (L.D.)

**Keywords:** camera network placement, coverage, particle swarm optimization (PSO), task constraints, camera constraints, scene constraints

## Abstract

As the use of camera networks has expanded, camera placement to satisfy some quality assurance parameters (such as a good coverage ratio, an acceptable resolution constraints, an acceptable cost as low as possible, *etc.*) has become an important problem. The discrete camera deployment problem is NP-hard and many heuristic methods have been proposed to solve it, most of which make very simple assumptions. In this paper, we propose a probability inspired binary Particle Swarm Optimization (PI-BPSO) algorithm to solve a homogeneous camera network placement problem. We model the problem under some more realistic assumptions: (1) deploy the cameras in the 3D space while the surveillance area is restricted to a 2D ground plane; (2) deploy the minimal number of cameras to get a maximum visual coverage under more constraints, such as field of view (FOV) of the cameras and the minimum resolution constraints. We can simultaneously optimize the number and the configuration of the cameras through the introduction of a regulation item in the cost function. The simulation results showed the effectiveness of the proposed PI-BPSO algorithm.

## 1. Introduction

Camera networks are used in many novel applications, such as video surveillance [1], room sensing [2], smart video conferencing [3], *etc.* There are some challenge issues in the study of camera networks, such as how to get an optimized camera network coverage, how to design a scalable network architecture, how to determine the trade-off between QoS requirements and energy costs [4], *etc.* Among the problems mentioned above, camera network coverage problem is a central issue, which has interested many researchers [5–9] and coverage rate is one of the most important performance metrics for camera network surveillance utility. Thus, determining the appropriate placement of the cameras to achieve the maximum amount of visibility becomes an important issue in designing camera network arrangements.

The camera network deployment problem can be defined as how to place the cameras in the appropriate places to maximize the coverage of the camera network under some constraints. The constraints can be categorized into three main types: task constraints, camera constraints and scene constraints. The task constraints include continuous tracking (enough overlap between cameras), people identification (image resolution and focus), complete coverage of the surveillance area (field of view of each camera) and so on. The camera constraints include the camera network type (a homogeneous or heterogeneous camera network), camera type (PTZ or static camera), camera intrinsic parameters (focus length, CCD size, *etc.*) and so on. The scene constraints include the surveillance area (2D or 3D, with or without holes, simple polygon or not), the positions where the camera network can be located (2D or 3D, in the walls, in the ceiling, at the same height or anywhere) and so on.

Since the camera network placement problem is a NP-hard combinatorial optimization problem [10], simple enumeration and search techniques will meet great difficulty in determining optimal placement configurations. There are many approximate techniques for solving optimal camera placement problems, such as the greedy based method, sampling based methodm *etc.* Zhao *et al.* have provided an excellent survey of the approximate techniques in [11]. Lately, some researchers have proposed some evolution-based optimization methods, such as PSO [12], BPSO-PI [13] and ABC [14].

There are some weaknesses in these results, mainly due to the overly simple assumptions used. From the perspective of the three constraints mentioned above, we give a brief explanation of the limitations of the earlier works. From the perspective of task and camera constraints, most of the works only consider the coverage of the area while the video resolution and focus are seldom considered; From the perspective of scene constraints, most of the scenes are modeled as a 2D case which is too simple to conduct the real camera network placement, or modeled as a 3D case which is too restrictive because in most of the cases we are only concerned with the surveillance plane area.

We give several examples. The surveillance area of [13] is modeled as a rectangle in the 3D cases while we know that in the real circumstances it is a trapezoid which is sensitive to the orientation of the cameras. The constraints in [14,15] only include the coverage rate (FOV is considered), while the resolution and focus are out of the scope of the articles.

**Figure 1.**
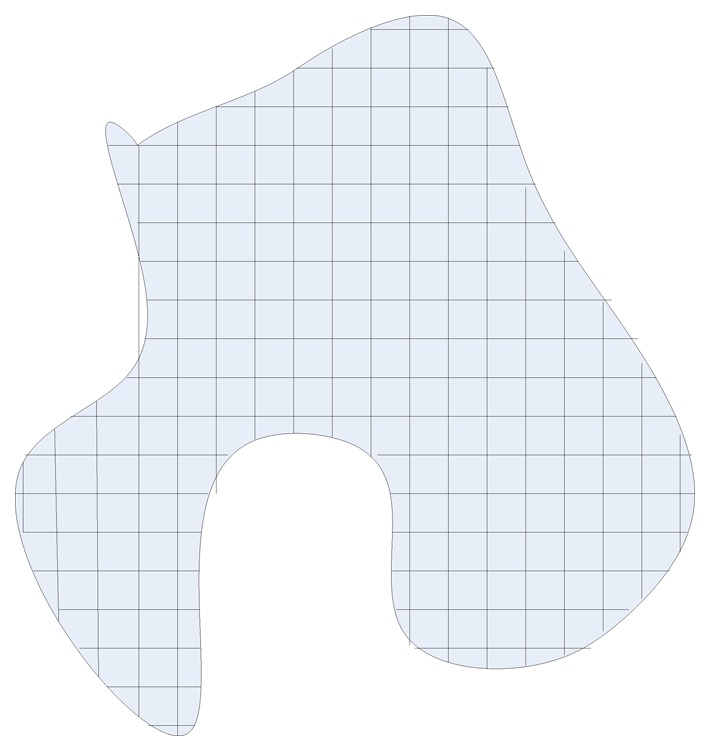
The surveillance plane area is divided into *n* grids no matter the shape of the area.

In this paper, we consider the deployment of homogeneous camera network in the 3D space to surveil a 2D ground plane. For simplicity considerations, the surveillance plane is modeled as a rectangle area which is not essential to our work. We separate the surveillance plane area into *n* grids, as illustrated in Figure 1. We assume that the probability of choose each grid is the same 1/*n* and the coverage ratio *p* can be determined by sampling as illustrated in the next section.

We take a more synthetic constraints set, including the surveillance video resolution, video focus, the camera field of view *etc.*, into consideration. Under the constraints, we propose a probability-inspired particle swarm optimization algorithm to get the optimized camera network placement configuration.

The main contributions of this paper can be summarized as follows:

- We consider a more realistic problem in that we deploy the cameras in a 3D space to surveil a plane area. Some of the previous works consider the problem in a 2D plane and the FOV of the camera is modeled as a sector which is too simple an assumption, while some works consider the problem in the 3D space and model the FOV of the camera as a cone which is too restrictive an assumption. We can get a more accurate result to solve the camera deployment problem in the 3D space to surveillance of a 2D plane and instruct the real life camera network placement;
- We take more constraints into consideration than others, including resolution, focus, FOV. Most of the previous works only consider the FOV of the camera to get a good coverage while the other constraints are important to get a good surveillance video;
- We propose a probability-inspired PSO algorithm to solve the camera network placement problem heuristically. In the algorithm, we introduce a regulated item in the fitness function to optimize the coverage ratio and the number of the cameras simultaneously. The experimental results show the effectiveness of the algorithm.

The rest of the paper is organized as follows: we review recent progress in camera network deployment in Section 2. In Section 3, we give the camera network placement problem from three perspectives. In Section 4, we propose a PI-BPSO algorithm and discuss the representation and fitness computation of particles in the algorithm. Simulation results are given in Section 5. We give the conclusions and discuss some future work in the last section.

## 2. Related Works

In Computational Geometry, there is a well-known problem called the Art Gallery problem (AGP) [16] and some of its variations which are very similar to the problem of camera placement in camera networks. The aim of the camera network deployment is to provide full coverage of the surveillance area with the minimal cost, and the aim of the AGP is to monitor an art gallery with the least number of guards located at different locations in order to make sure that every point in the museum is seen by at least one guard. The difference between the two problems is mainly attributable to the assumption on the ability of the “guard” as the AGP assumes the guards have unlimited field of view, infinite depth of field, and have infinite precision and speed, while the cameras in the real world don't have these abilities. Even if we make the unrealistic assumption of the ability of the guard, the problem is still proved to be NP-hard [17,18]. Though the AGP and its variants can't give an exact answer to the camera network placement problem, they do give some insights into the problem, the visibility graph and the lower bound of the guard numbers. There are some good results in the AGP field which we will not introduce here in detail. We refer the reader to an excellent book on the topic [16].

The other interesting research problem related to the camera network placement problem is the wireless sensor network (WSN) placement problem [4,7,19–22]. To be strict, the camera is known as a visual sensor, but because the camera is a kind of directional sensor, this leads to some differences between the two disciplines. We refer the reader to the review [6] about the WSN placement problem.

Researches on the camera network placement problem can be divided into two disciplines, one is the coverage problem and the other is the optimization problem. The two problems can be related through the optimization framework issued by Zhao *et al.* [11], where they present that the camera network placement can be divided into two broad categories, the MIN and FIX problems. The MIN problem is to find the minimum cost cameras to satisfy the minimum coverage ratio, and the FIX problem is to find the maximum the coverage subject to a fixed number of cameras.

The visual coverage of the camera network describes what can be seen and what can't in the surveillance area. It is so fundamental to many computer vision tasks that different works have suggested different visual coverage models according to the different surveillance tasks.

The standard coverage model is defined as the area of the surveillance area. It can be classified as two cases, from the perspective of the area and the camera. From the perspective of the area [13,23], the surveillance area is discrete into some grid and if the center point of a grid can be seen from a camera (under some limitation such as resolution and DOF *etc.*), then we say that the grid can be seen from the camera. From the perspective of the camera [24,25], the area that a cameras can monitor is determined by the camera's FOV, DOF, resolution and the camera network placement problem is turned into a set cover problem. To get a continuous consistently labeled trajectory of the same object, Yao *et al.* [26] add handoff rate analysis to the standard coverage models. To improve the full coverage of events and objects/events recognition, Newell *et al.* [27] give an multi-perspective coverage (MPC) model where the coverage is calculated based on the ω-perspective coverage (the number of perspectives that cover the event). Based on the MPC model, Yildiz *et al.* [8] give an angular coverage model where the coverage of an object is defined as the object can be seen from different perspectives that span 360°. We refer the reader to the excellent survey [5]. Most of the work model the area as a plane area and the camera is placed in the plane which is too simple to guide the real camera network placement.

As we have stated that the camera network placement problem is NP-hard, researchers have put forward various approximate optimization algorithms to solve the problem [11,13–15]. Chrysostomou *et al.* [14] propose a bee colony algorithm as the optimization engine to determine the minimum possible cost (minimum number) of cameras to cover the given space under some camera placement constraints such as geometrical, optical, as well as reconstructive limitations and this delivers promising preliminary results. Morsly *et al.* [13] propose a Binary Particle Swarm Optimization Inspired Probability (BPSO-IP) algorithm as the optimization engine to ensure the accurate visual coverage of the monitoring space with a minimum number of cameras. The authors also give a detailed comparison between the BPSO-IP algorithm and other evolutionary-like algorithms such as BPSO, Simulated Annealing (SA), Tabu Search (TS) and genetic techniques based algorithms to solve the camera network placement problem. Lee *et al.* [15] give a generic algorithm as the optimization engine to solve the camera network placement problem which is modeled as a multi objective optimization problem. Zhao *et al.* [11] put forward a framework to compare the accuracy, efficiency and scalability of the greedy-based method, heuristics-based method, sampling-based method and LP and SDP relaxation-based method.

## 3. Problem Definition and System Model

We put forward an optimal camera network placement problem to satisfy the need of different surveillance tasks on a specific surveillance area. The problem can be modeled as a multi objective optimization problem that must satisfy multiple constraints. In this work, we are interested in the static camera network placement problem, where the objective is to determine the number of cameras, their positions and poses for an rectangle surveillance area, given the intrinsic parameters of the cameras (such as focal length, the diameter of the lens's aperture *a*, the minimum dimension of a camera pixel *c*, the resolution R, *etc.*) and a set of task-specific constraints (such as the resolution constraints, the focus constraints and visibility constraints *etc.*). Commonly, this camera network placement problem takes place off-line to support the task-specific requirements of on-line computer vision surveillance systems. But occasionally we shall adjust the layout of the camera network on-line to support the different surveillance tasks.

**Figure 2.**
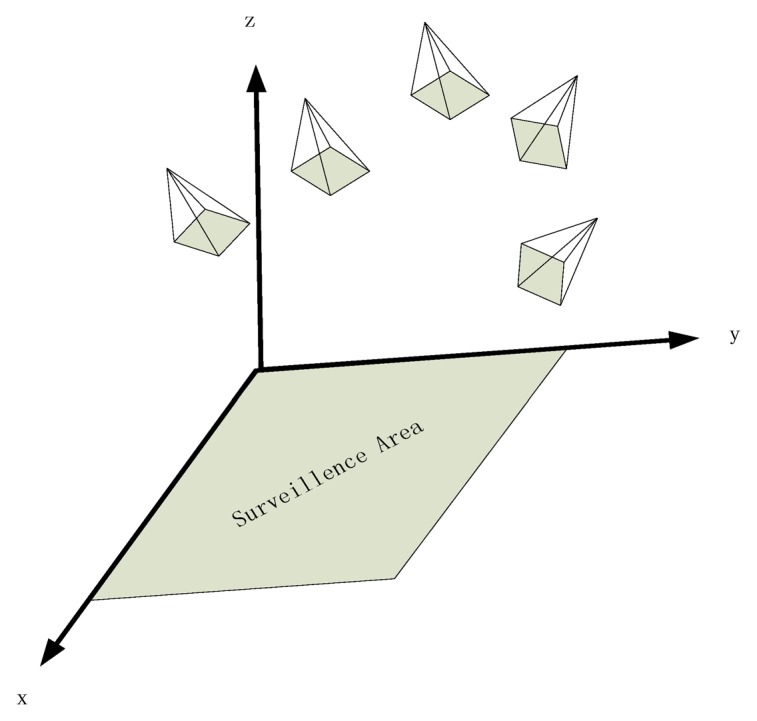
The coordinate frame we establish to model the surveillance scene where the ground plane (the surveillance area) is modeled as the *XOY* plane and the anti-gravity direction is modeled as the *Z* direction.

In the real world circumstances, we often layout some linked cameras to surveillance a square which is the main subject of this article. We establish a world frame as that: we model the surveillance square as the *XOY* plane and the upward direction as the *Z* axis which is shown in Figure 2.

### 3.1. Camera Modeling

We assume that the various cameras used in the layout share the same intrinsic parameters, such as the resolution of the camera is *R*(*R_h_*,*R_v_*), the horizontal and vertical dimensions of the size of the Coupled Charge Detector (CCD) element is *s*(*h*,*w*), the focal length of the camera is *f*, *etc.* In our work we are mainly interested in the video resolution constraints, which is very common in surveillance tasks such that we can identify some persons in the surveillance video, and the focus constraints which are necessary to get a clear surveillance video.

#### 3.1.1. Modeling a Camera's FOV (Field of View) in 3D Space

**Figure 3.**
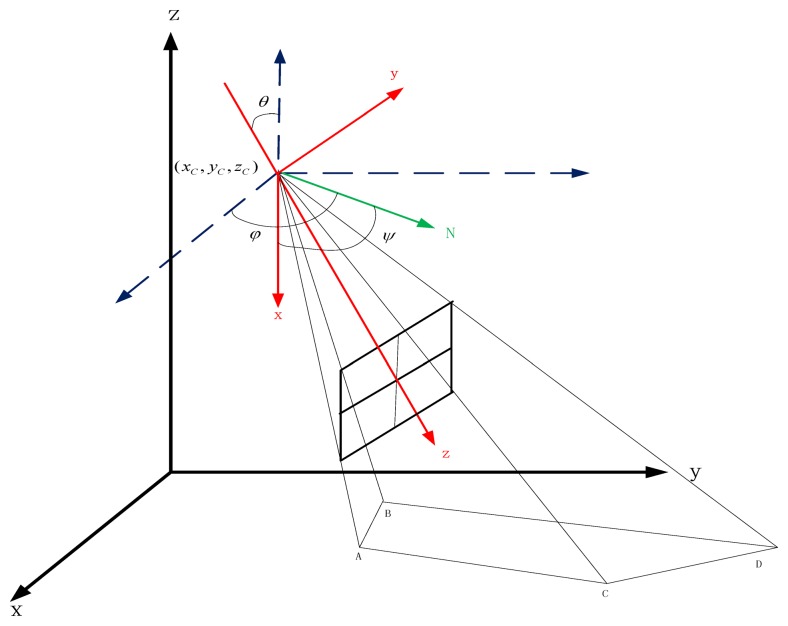
The FOV of the camera in 3D space and the camera's yaw, pitch, roll angles which is shown as (*ϕ*, *θ, ψ*). The vector *N* is the intersect of the *XOY_camera_* plane and the *XOY_world_t(C)_* (the coordinates that transform the world coordinate to the point *C*) plane.

We use a pyramid to represent the camera's FOV, which is shown in Figure 3. We are interested in the homogeneous camera network placement problem in this article. We set *K* as the intrinsic parameter matrix of the cameras in the network. The camera's position C(*x_C_*,*y_C_*,*z_C_*) and rotation *R*(*ϕ*, *θ, ψ*) describe the camera's extrinsic parameters where *R*(*ϕ*, *θ, ψ*) is the Euler angles representation of the rotation matrix *R* [28] and (*ϕ*, *θ, ψ*) represents the camera's yaw, pitch and roll angles respectively (Figure 3) and we have:

(1) $$R=\left[\begin{array}{ccc} \sin\psi & -\cos\psi & 0\\ \cos\psi & \sin\psi & 0\\ 0 & 0 & 1 \end{array}\right]\;\left[\begin{array}{ccc} 1 & 0 & 0\\ 0 & \cos\theta & \sin\theta \\ 0 & -\sin\theta & \cos\theta \end{array}\right]\;\left[\begin{array}{ccc} -\sin\phi & \cos\phi & 0\\ -\cos\phi & -\sin\phi & 0\\ 0 & 0 & 1 \end{array}\right]$$

For the surveillance application, we desire that the angle between the object and the direction of the camera is less than a constant angle *θ*_0_ (which is critical for feature point extraction and the other applications), such as 60°. We assume that the object in the surveillance video is vertical to the ground (which is almost true because Pisa tower is seldom), then we get a constraints on the (*ϕ*, *θ, ψ*) that *θ* ≤ *θ*_0_. As Figure 3 shows, the surveillance area of the camera is $\bar{\text{ABCD}}$, and the coordinates of the 4 vertexes can be determined by:

(2) $$\begin{array}{c} \left[\begin{array}{cccc} -\frac{h}{2} & \frac{h}{2} & \frac{h}{2} & -\frac{h}{2}\\ -\frac{w}{2} & -\frac{w}{2} & \frac{w}{2} & -\frac{w}{2}\\ 1 & 1 & 1 & 1 \end{array}\right]=K[\begin{array}{cc} R & -\bar{C} \end{array}]\left[\begin{array}{cccc} x_{1} & x_{2} & x_{3} & x_{4}\\ y_{1} & y_{2} & y_{3} & y_{4}\\ 0 & 0 & 0 & 0\\ 1 & 1 & 1 & 1 \end{array}\right]\\ =K[\begin{array}{ccc} R_{1} & R_{2} & -\bar{C} \end{array}]\left[\begin{array}{cccc} x_{1} & x_{2} & x_{3} & x_{4}\\ y_{1} & y_{2} & y_{3} & y_{4}\\ 1 & 1 & 1 & 1 \end{array}\right]\\ =P\left[\begin{array}{cccc} x_{1} & x_{2} & x_{3} & x_{4}\\ y_{1} & y_{2} & y_{3} & y_{4}\\ 1 & 1 & 1 & 1 \end{array}\right] \end{array}$$

where *h*, *w* is the CCD height and width, *K* is the intrinsic matrix of the camera and (*x_i_*, *y_i_*) 1 ≤ *i* ≤ 4 is the coordinates of the vertexes of $\bar{\text{ABCD}}$. We can transfer the above equation to a more simple one:

(3) $$\left[\begin{array}{cccc} x_{1} & x_{2} & x_{3} & x_{4}\\ y_{1} & y_{2} & y_{3} & y_{4}\\ 1 & 1 & 1 & 1\\ 1 & 1 & 1 & 1 \end{array}\right]=P^{†}\left[\begin{array}{cccc} -\frac{h}{2} & \frac{h}{2} & \frac{h}{2} & -\frac{h}{2}\\ -\frac{w}{2} & -\frac{w}{2} & \frac{w}{2} & -\frac{w}{2}\\ 1 & 1 & 1 & 1 \end{array}\right]$$

If we know that a camera's configuration (*R* –*C̅*), we can get the coordinates of the four vertices of the quadrangular surveillance area in the plane. Then we can determine whether the point *P* in the plane can be covered by the configuration use $P\in \bar{\text{ABCD}}$.

#### 3.1.2. Modeling the Resolution Constraints in 3D Space

**Figure 4.**
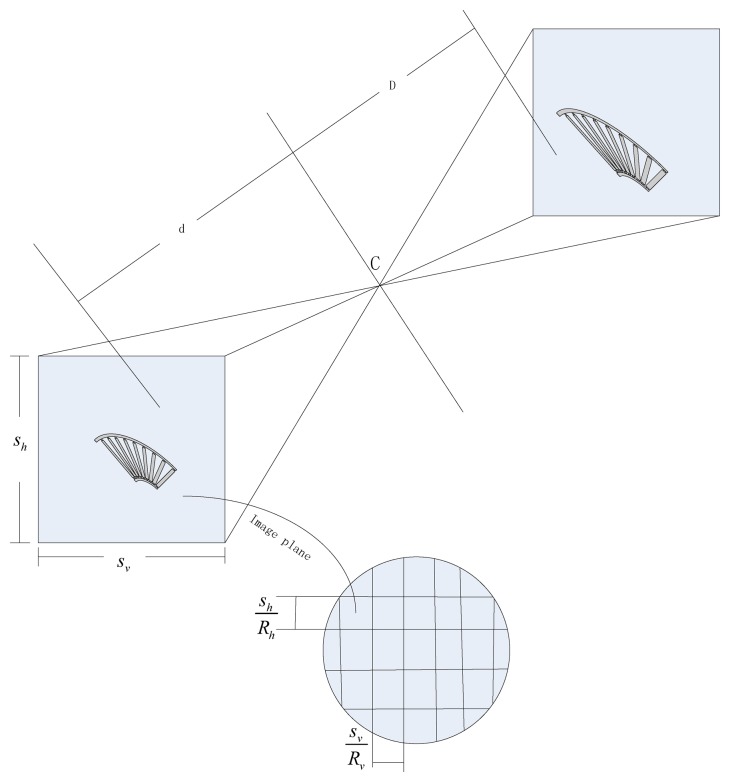
Camera and spatial resolution for the camera.

In this section we describe the relationship between the surveillance video resolution constraints and the camera's position. For a specific surveillance camera, the required resolution provides an upper bound on the distance between the camera and the surveillance area. Figure 4 illustrates the image process of an object *S* lying at distance *D* from the lens center, where the distance between the image and the lens center is *d*. There is a relationship between *d*, *D* and the lens focal length *f* by the Gaussian lens equation:

(4) $$\frac{1}{d}+\frac{1}{D}=\frac{1}{f}$$

The surveillance video resolution of object *S*, described as the pixels per unit length, depends on the direction in which it is measured. It is easy to see that the maximum resolution occurs along the rows or columns of pixels and the minimum occurs along the diagonal of each pixel. In consideration of simplicity, we assume that the desired resolution constraints is *α* pixels per unit length corresponding to the diagonal of each pixel, then:

(5) $$\frac{1}{D_{\text{Max}}}=\frac{\alpha (\sqrt{{\left(\frac{h}{R_{h}}\right)}^{2}+{\left(\frac{w}{R_{\nu }}\right)}^{2}})}{d}$$

The distance between the position of the camera len's center and the surveillance area *D* must satisfy:

(6) $$D\leq D_{\max}$$

#### 3.1.3. Modeling the Focus Constraints in 3D Space

**Figure 5.**
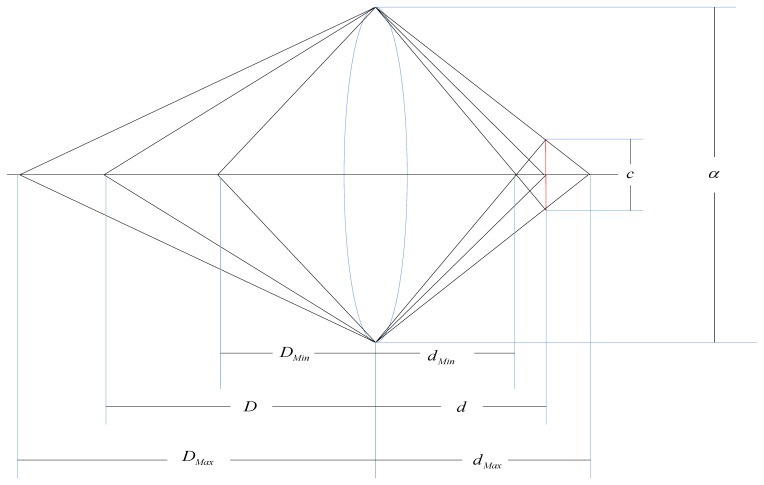
DOF for the camera.

In this section, we determine the constraints on the camera's viewpoints when we request that all the points of the surveillance area must be sharp (in focus) in some surveillance camera's FOV. For any camera, there is only one plane on which the camera can precisely focus, and the point object in any other plane is imaged as a disk (known as the blur spot) rather than a point. When the diameter of blur spot is sufficiently small, the image disk is indistinguishable from a point. The diameter of the blur spot is known as the acceptable circle of confusion, or simply as the circle of confusion (*CoC*). We can easily induce that there is a region of acceptable sharpness between two planes on either side of the plane of focus which is illustrated in Figure 5. The region is known as depth of field (DOF).

Here we assume the *CoC* is the minimum dimension of a camera pixel *c*, The focus distance is *D*, the lens's focal length *f*, the diameter of the lens's aperture *α*, relative aperture (f-number) of lens *N*. We can determine the maximum distance *D_Max_*, and the minimum distance *D_Min_* as follows [29]:

(7) $$D_{\text{Max}}=\frac{Df^{2}}{f^{2}-Nc(D-f)}$$

(8) $$D_{\text{Min}}=\frac{Df^{2}}{f^{2}-Nc(D-f)}$$

(9) $$N=\frac{f}{\alpha }$$

If we want the video to be sharp, then:

(10) $$D_{\text{Min}}\leq D\leq D_{\text{Max}}$$

**Figure 6.**
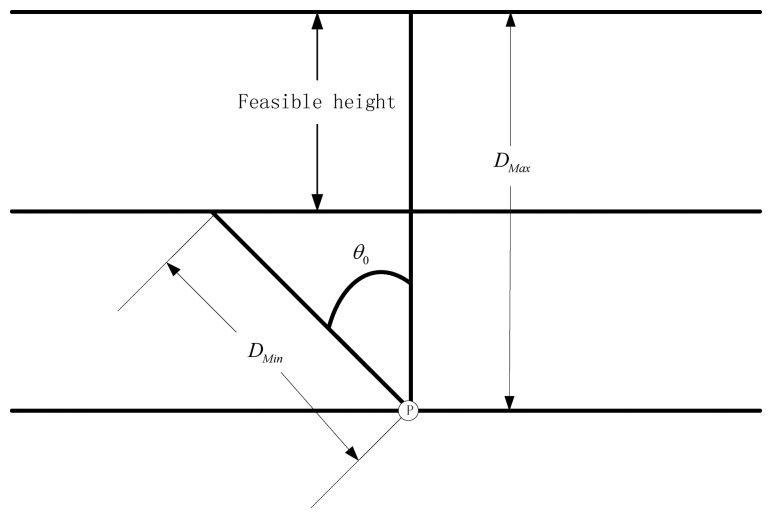
The feasible height of the cameras. We can see that the point *P* will be not sharp if the height of the camera's height *H* don't satisfy *D_Min_Cosθ*_0_ ≤ *H* ≤ *D_Max_*. That is to say that *D_Min_Cosθ*_0_ ≤ *H* ≤ *D_Max_* is a necessary condition for the point *P* in sharp. *θ*_0_ is the biggest feasible angle between the direction of the camera and the object which is discussed above.

Then we have that the height of the camera must satisfy (illustrated in Figure 6):

(11) $$D_{\text{Min}}Cos\theta_{0}\leq H\leq D_{\text{Max}}$$

### 3.2. Space Modeling

In theory, cameras can be located anywhere in the space since the camera position variables *x_C_*, *y_C_*, *z_C_* and the pose variables *φ*, *θ* and *ψ* are all continuous variables. In practice, we ordinarily restrict the selection of the cameras' location and pose in a discrete parameter space which is determined by the spatial sampling in the continuous parameter space. So we can transform the continuous optimization problem to a discrete optimization problem. We should state that as the spatial sampling frequencies *f_x_*, *f_y_*, *f_z_*, *f_φ_, f_θ_*, and *f_ψ_* → ∞, the approximated discrete solution converges to the continuous-case solution.

In the real situation, there are some other different constraints on the allowed deployable area such as the constraints that the cameras must be located on the walls of the indoor surveillance environment or the constraints that the cameras must be located at some restricted height for the consideration of their management, but in this article we only restrict the position of the cameras according to the constraints of surveillance video resolution, FOV (field of view) and DOF (depth of field), which are described in the previous section.

### 3.3. Camera Network Placement Problem Modeling

We can model the camera network placement as an optimization problem which is defined as maximization of some utility function given some cameras and task constraints. From the definition of the model, we can analyze the problem from four perspectives: the utility function (or cost function), the task constraints, the space constraints and the camera's parameters. Let *T* be the given task and let *C_T_* be the set of all constraints imposed by the task *T* as: quality of service constraints like resolution constraints and focus constraints, square coverage constraints, camera network overlap ration constraints *etc.*

Let the vector *C*(*C^I^*,*C^E^*) represents the camera's intrinsic and extrinsic (position and pose) parameters. The problem is to find where to layout the set of cameras in the specific area *A* to minimize the cost function under the set of constraints *C^T^*:

(12) $$\arg\min_{C}G(C)\;\text{where}\;C\in \text{A}\cap {\text{C}}_{T}$$

Where *C* = (*C_i_*,*i* = 1,2,…,*N*) is the cameras we want to place in the surveillance area *A*, *G*(*C*) is the cost function.

In this work we are mainly interested in the 2D plane surveillance by a camera network located in a 3D environment. In this special surveillance scene, we want to place the minimum number of cameras in the optimal locations (position and pose) to maximize the visual coverage under the surveillance task constraints. Based on the assumptions we make above, we give the following specific model to solve this visual sensor placement.

Suppose that we have *N* cameras *C* = (*C_i_*,*i* = 1,2,…,*N_C_*) in the network, and the positions and poses that we can place our cameras are *S* = (*S_i_*,*i* = 1,2,…,*N_S_*) (which are determined by the space modeling we discuss in the section above), and the surveillance area *A* is modeled as a surveillance space *T* = (*T_i_*,*i* = 1,2,…,*N_T_*). The surveillance space has different forms according to the different tasks. In the situation we are interested in this article, we reduced the area to a rectangular grid and choose the middle point of each grid (*T_i_*,*i* = 1,2,…,*N_T_*) as the representation of the surveillance area.

We define a binary variable {*b_i_*_,_*_j_* :*i* = 1,…,*N_S_,j* = 1,…,*N_C_*} to represent the camera network configuration as follows:

*b_i_*_,_*_j_* = 1 if a camera *C_j_* is placed in a location *S_i_*
*b_i_*_,_*_j_* = 0 otherwise

We define a binary variable {*x_i_*_,_*_j_* :*i* = 1,…,*N_T_,j* = 1,…,*N_C_*} to represent the surveillance utility of the camera network.

*x_i_*_,_*_j_* = 1 if a camera *C_j_* can see the object *T_i_*
*x_i_*_,_*_j_* = 0 otherwise

Based on the notations we define above, we define the utility function *G*(*C*) as:

(13) $$G(C)=\mu (C)+\lambda f(N_{C})$$

where *μ*(*C*) is the surveillance utility function of the camera network which is increase as the number of the cameras increase. In our experiment, we set *μ*(*C*) = *p* where *p* is the ratio of the surveillance area:

(14) $$p=\frac{\sum_{i=1}^{N_{T}}\max_{j}(x_{i,j})}{N_{T}}$$

When the number of grids in the surveillance area is too large, then we can apply a sample method to determine the coverage. When we choose *m* grids to check if they are covered by the camera network and *k* grids is covered then we can see that the coverage rate is:

(15) $$p=\frac{k}{m}$$

*f*(*N_C_*) is a regulation item which decreases as the number of the cameras increases, so it can be a punitive item to the number of the cameras. In our experiment, we set $f(N_{C})=\frac{1}{N_{C}}$.

Based on the augment above, we turn the camera network placement problem to an optimization problem as follows:

(16) $$\arg\max_{C}(\frac{\sum_{j=1}^{N_{T}}\max_{i}(x_{ij})}{N_{T}}+\lambda \frac{1}{N_{C}})$$

Satisfies:

$\sum_{j=1}^{N_{C}}b_{i,j}\leq 1$ one location only has one camera
$\sum_{i=1}^{N_{S}}b_{i,j}\leq 1$ a camera can only be placed in one state (position and pose)
$\frac{\sum_{j=1}^{N_{T}}\max_{i}(x_{ij})}{N_{T}}\geq p_{0}$ the surveillance ration must exceed *p*_0_.

## 4. Optimization Method

Evolutionary computation technique, motivated by the evolution of Nature, is a powerful tool to approximately solve many NP-hard problems. There are many kind of evolution computing methods, such as genetic algorithm (GA) [30], differential evolution (DE) [31], artificial bee colony (ABC) [32,33]. The differences between different evolution algorithms mainly result from the different observations on Nature and the methods used to get new solutions from the old hypothesis. Among the various evolutional computing methods, Particle Swarm Optimization (PSO) [34] inspired by the manner that a flock of birds or fishes exhibit a coordinated collective behavior during the travel, is an optimization tool used to deal with various optimization problems [13,35].

The PSO algorithm consists of a population of agents called particles, each of which is a potential solution to the optimization problem. The particle *i*, has a memory to record the current (time *t*) position (solution) $x_{i}^{t}$, the previous personal best position $pb_{i}^{t}$ and a velocity $\nu_{i}^{t}$ at which speed the particle fly to the next position. We assume that the global best position of the group of particles is *gb^t^* until time *t*. The position and velocity of each particle in standard PSO algorithm are updated as the followed equations:

(17) $$x_{i}^{t+1}=x_{i}^{t}+\nu_{i}^{t}$$

(18) $$\nu_{i}^{t}=\overset{\text{inertia}}{\omega \nu_{i}^{t-1}}+\overset{\text{personal}}{\overset{︷}{\varphi_{1}U_{1}^{t}(pb_{i}^{t}-x_{i}^{t})}}+\overset{\text{society}}{\overset{︷}{\varphi_{2}U_{2}^{t}(pb^{t}-x_{i}^{t})}}$$

where *ω* is the inertia weight, *φ*_1_ and *φ*_2_ are the weights of the personal influence and the society influence, $U_{1}^{t}$ and $U_{2}^{t}$ are two random numbers uniformly distributed in the interval [0,1]. There are many variants of the standard PSO algorithm, and the main differences between them lies in the velocity update strategy used.

Despite the different form of the various PSO algorithms that can be used, the main task during the solving process is to determine the representation of the particles and the fitness of each particle. That is to say, to solve the camera network placement with the PSO approach, we should build a mapping between the camera network deployment solution to the particle state and calculate the fitness of each particle's state. Because we represent the solution space in a discrete space, we must have a mechanism to transform the new position of the particle to a legal place. Like any optimization method, the initialization is very important for the convergence speed and solution quality. We will illustrate the important factors above in the next sections.

### 4.1. Representation

In this section, we describe the state representation of the camera network placement problem. Each of the PSO particles is represented as a *f_x_* × *f_y_* × *f_z_* × *f_φ_* × *f_θ_* × *f_ψ_* dimensional array, where each column is a binary value representing the position and the orientation of the camera to be deployed. From the definition, we know that the number of 1 in the array represent the number of the cameras deployed in the network. The PSO population of the particles can be set to a const, such as 10,000.

Next we show how to represent a camera network placement problem as an example. Suppose that we deploy four cameras to surveil a rectangular area and the cameras can be placed in a $\overset{\text{Position}}{\overset{︷}{2\times 2\times 1}}\times \overset{\text{Orientation}}{\overset{︷}{2\times 2\times 1}}$ grid in the 3D space where *f_z_* = 1 represents that we set the cameras in the same height above the surveillance plane in the example and *f_ψ_* = 1 represents that we neglect the influence of the rotation of the cameras across the axis *Z* in the example. We can simplify the particle state space to a $\overset{\text{Position}}{2\times 2}\times \overset{\text{Orientation}}{2\times 2}$ space.

**Figure 7.**
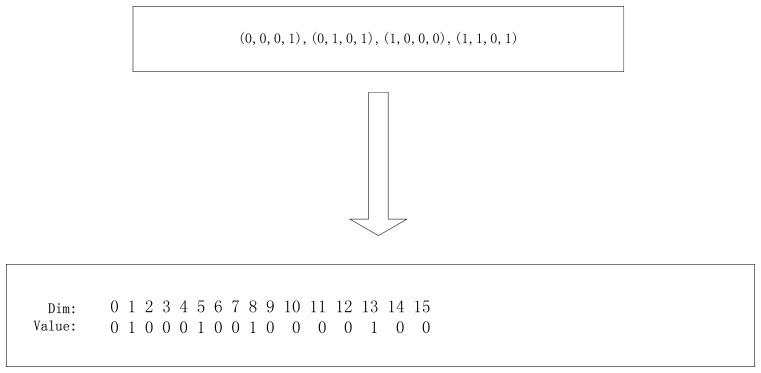
A particle state representation for a camera network placement example where we can see that the number of 1 in the particle state is 4.

Figure 7 shows a mapping between one camera network placement instance to a particle's state in the PSO state space where each tuple represent the corresponding position and pose of a deployed camera on the 3D grid. For example, the camera state (0,0,0,1) can be transferred to the position 0 × 2^3^ + 0 × 2^2^+ 0 × 2^1^ + 1 × 2^0^ = 1 of 1 in the particle space, and the other camera state can also be transformed to the position of 1 in the particle state.

### 4.2. Fitness Assignment

**Figure 8.**
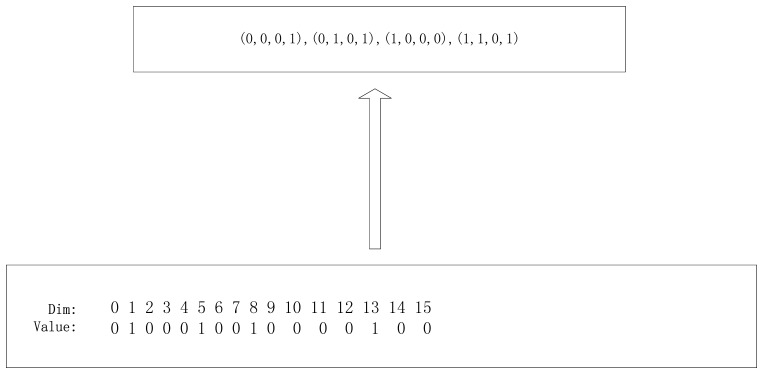
A camera network placement strategy for a particle state which is a inverse process of the particle representation.

The fitness of each particle is calculated according to the cost function *G*(*C*) of the camera network placement problem. At first we should transfer the particle's state to a camera network placement solution which is a inverse problem of the particle representation which is shown in Figure 8.

Then we can get the fitness of the camera network configuration.

### 4.3. Flowchart of the Proposed PI-BPSO Algorithms

**Figure 9.**
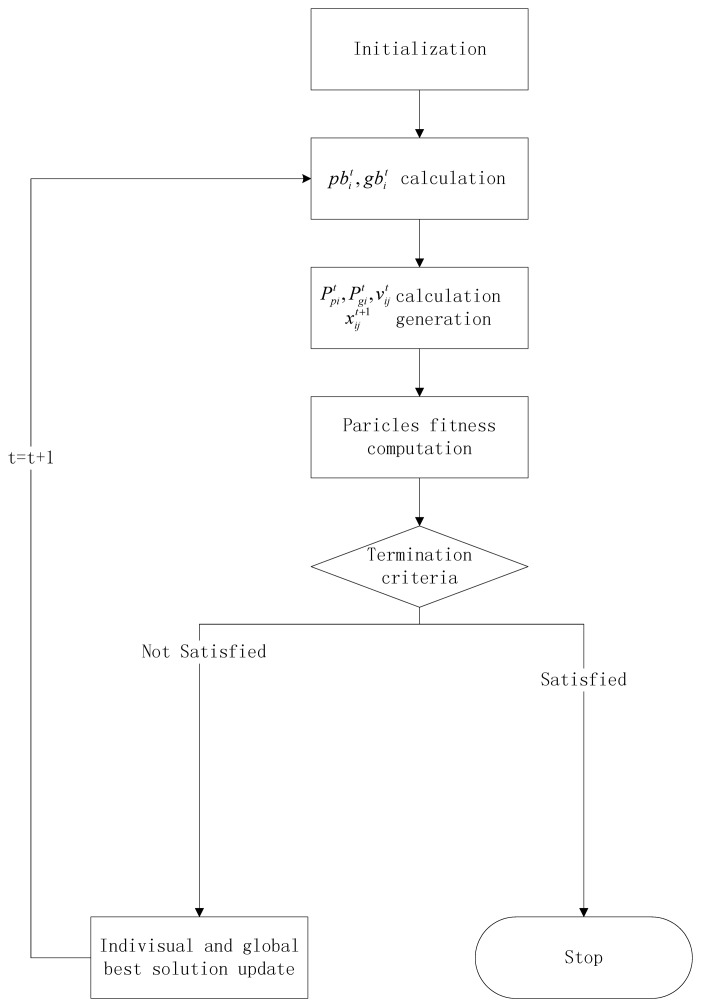
PI-BPSO flowchart. We should point out that we should calculate both the personal best and global best of the particles $p_{pi}^{t}$, $p_{gi}^{t}$ to compute the velocity of the particle.

The flows of most PSO algorithms are very similar, that is a four step iterations of “initialization, evaluate, update, stop”. The main difference between them is the strategy of new particles generation and the update strategy. In this work, we propose a probability induced binary particle swarm optimization algorithm (PI-BPSO) which is a extension of the algorithm propose in [13]. In PI-BPSO, the bit value of each particle's state is determined by the “or” value of its current value (current state *x_ij_*) and the probability of being “1” (velocity *ν_ij_*) which is updated according to the information sharing mechanism of PSO. The main steps of PI-BPSO can be described as follows (see Figure 9).

***Step 1: Initialization.*** Setting the size of the population of the PSO and create the population of particles which is encoded as the position and orientation of the cameras in the network.
***Step 2: Calculation.*** Calculate each particle *i*'s best state $pb_{i}^{t}$ and the global best state *gb^t^* of particles until time *t*:
  (19) $$pb_{i}^{t}=\arg\max_{t}G(C_{i})$$
  (20) $$gb^{t}=\arg\max_{t}pb_{i}^{t}$$
***Step 3: New particle generation.*** Generate the new position of the particle *i* according to the probability $p_{pi}^{t}$, $p_{gi}^{t}$ step by step.

First we calculate the probability $p_{pi}^{t}$, $p_{gi}^{t}$as follows:

(21) $$p_{pi}^{t}=\frac{1}{1+(G(C_{pi}^{t})-G(C_{i}^{t}))}$$

(22) $$p_{gi}^{t}=\frac{1}{1+(G(C_{g}^{t})-G(C_{i}^{t}))}$$

where $G(C_{pi}^{t})$ is the utility when the particle *i* in its best position until time *t*, $G(C_{i}^{t})$ is the utility when the particle *i* in time *t*, $G(C_{g}^{t})$ is the maximum utility of all the particles until time *t*.

Secondly, we generate two uniformly distributed random numbers $r_{ijp}^{t}$, $r_{ijg}^{t}$ and compare them with the probability $P_{pi}^{t}$, $P_{gi}^{t}$ to determine the personal velocity and society velocity as follows:

(23) $$v_{ij}^{pt}=\{\begin{array}{l} 0\ldots r_{ijp}^{t}<P_{gi}^{t}\\ 1\ldots \text{otherwise} \end{array}$$

(24) $$v_{ij}^{gt}=\{\begin{array}{l} 0\ldots r_{ijg}^{t}<P_{gi}^{t}\\ 1\ldots \text{otherwise} \end{array}$$

Thirdly, we determine the velocity at time *t*:

(25) $$a=\varphi_{1}\nu_{ij}^{pt}+\varphi_{2}\nu_{ij}^{gt}\;\text{where}\;\varphi_{1}+\varphi_{2}=1$$

(26) $$\nu_{ij}^{t}=\{\begin{array}{l} 0\ldots r_{ij\nu }^{t}\leq a\\ 1\ldots \text{otherwise} \end{array}$$

Fourthly, we determine the new state of the particle $i:x_{ij}^{t+1}=x_{ij}^{t}⊕\nu_{ij}^{t}$, where ⊕ is a logical operator we define as follows: If $\nu_{ij}^{t}=1$, $x_{ij}^{t+1}=x_{ij}^{t}$ else $x_{ij}^{t+1}=not(x_{ij}^{t})$ which is different from [13] where the operator is “OR”. In the work of [13], when one position is assigned to be “TRUE”, then it will almost always be assigned a camera there, we think this is unreasonable in some sense. We should explain one case clearly. If *x_ij_* = 1 for all *j*, the fitness of the configuration will not be the best particle candidate (because the regulation item in the utility function) and the velocity will have great probability to be zero. Considering the update strategy in our algorithm, some position of the particle will be set to “0” with great probability. So the optimization process will not halt.

***Step 4: Particle fitness computation.*** Compute the fitness of the particles according to its new positions $x_{i}^{t}$.
***Step 5: Decide whether to update or stop according to the terminate criteria.*** Determine whether the terminate criteria is satisfied (the upper limit of the iteration times or the accuracy precision). If the terminate criteria are satisfied, then output the global best particle, else update the new personal best solution of each particle and the global best particle and goto the next iteration.

In the algorithm aspect, the main difference between our algorithm and [13] is that we distinguish the person influence and the society influence which are very important for the success of the PSO algorithms while [13] only gives a update strategy which ignores the two influence factors.

There is another key difference between the two methods: PI-BPSO uses a regulation item in the cost function to determine the appropriate number of cameras while BPSO-PI [13] doesn't consider the issue in the article. The benefit of involving the number of cameras in the cost function is evident that is when the benefit of increasing of the number of cameras can't cover the cost of the increase of the number of the cameras, we should stop increasing the number of cameras. We think that this intuitive idea will help reach get the camera network placement solution more easily.

## 5. Simulation Results

Most of the constrained optimization problems solved by PSO [13,36] apply some kind of feasibility preserving strategy to handle the constraints, which means that we only preserve the particles that satisfy all the constraints mentioned above. Through the strategy we transfer the constrained optimization problem to an unconstrained ones.

For the problems in this article, we apply the following strategy:

(1) During the initialization stage, all particles are started with feasible solutions;
(2) During the updating stage, only the feasible particles are kept in their memories.

To evaluate the effectiveness and reliability of the proposed algorithm, we adopted the following experiment configuration:

(1) The surveillance area is a 50 m × 50 m square;
(2) The camera intrinsic parameter is $K=\left[\begin{array}{ccc} 4.8 & 0 & 0\\ 0 & 4.8 & 0\\ 0 & 0 & 1 \end{array}\right]$;
(3) The minimum resolution requirement is *α* = 1 pixel/cm (for face recognition, a face is imaged as a 20 × 20 square);
(4) The CCD size is 1/4^″^ (3.2 mm × 2.4 mm) and the resolution of the camera is 1,024 × 768;
(5) The Euler angle of the camera is restricted in the following range: $\frac{2\pi }{3}\leq \theta \leq \frac{3\pi }{4}$.

We do two kinds of experiments. One kind of experiment is to determine the influence of the different sampling frequencies on the solution of the camera network deployment problem; the second kind of the experiment is to determine the influence of the *λ* parameters on the cost functions. We set the population size at 20 and the maximum number of iterations in the experiments is 10,000.

**Figure 10.**
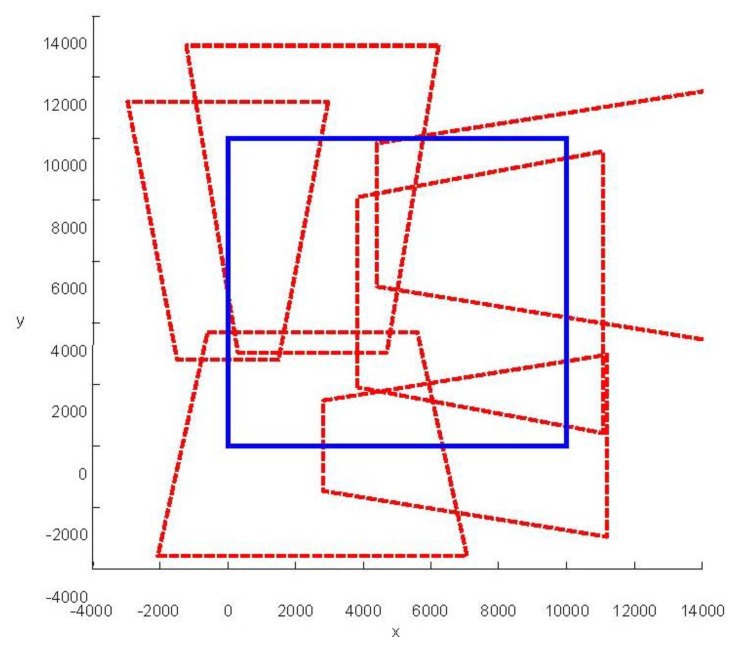
Optimal placement of cameras (six cameras). The surveillance area is 10 m × 10 m, *f_x_* = 4, *f_y_* = 4, *f_z_* = 2, *f_φ_* = 4, *f_θ_* = 2, *f_ψ_* = 4, *λ* = 1.

**Figure 11.**
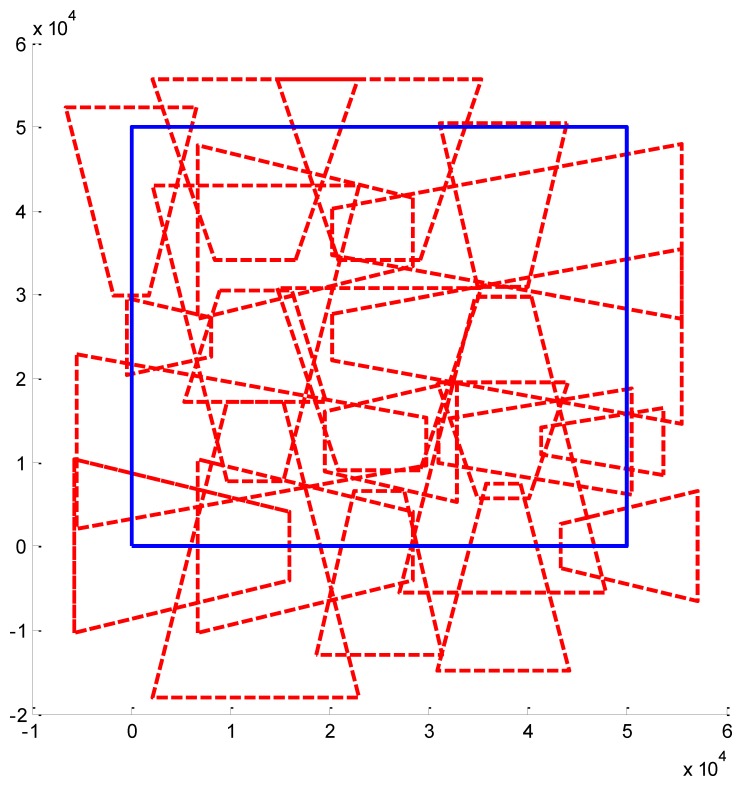
Optimal placement of cameras (24 cameras). The surveillance area is 50 m × 50 m, *f_x_* = 4, *f_y_* = 4, *f_z_* = 2, *f_φ_* = 4, *f_θ_* = 2, *f_ψ_* = 4, *λ* = 1.

**Figure 12.**
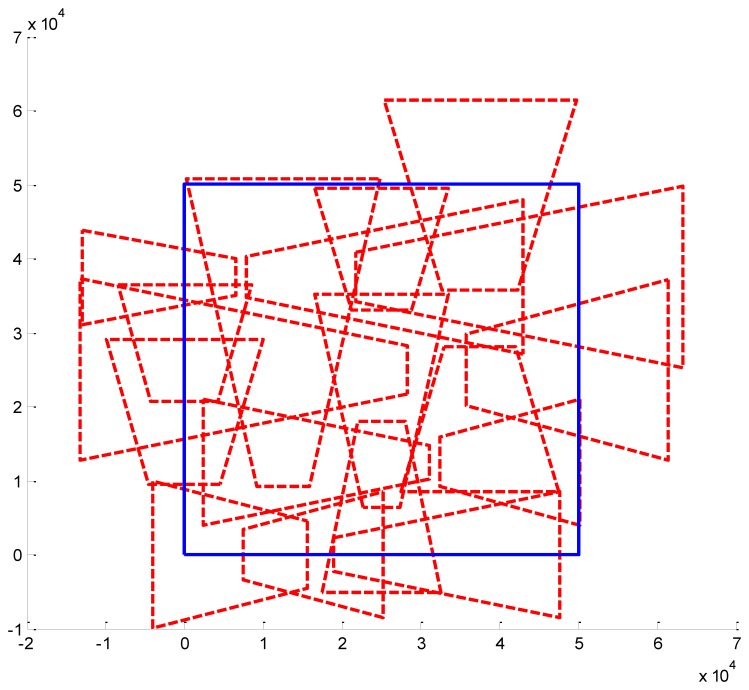
Optimal placement of cameras (16 cameras). The surveillance area is 50 m × 50 m, *f_x_* = 4, *f_y_* = 4, *f_z_* = 4, *f_φ_*=4, *f_θ_* = 4, *f_ψ_* = 4, *λ* = 1.

We present some results obtained by the algorithms (Figures 10, 11 and 12). In the figures, blue bold line represents the area that needs to be surveilled and the red dash lines represent the FOV of the cameras. In Figures 11 and 12, we choose the same surveillance area and choose different sample frequencies for the pose parameters. In Figure 11, the parameters is (*f_x_* = *f_y_* = *f_φ_* = *f_ψ_* =4, *f_z_* = *f_θ_* = 2), the fitness of the surveillance is 0.9785, the number of the surveillance cameras is 24; while in Figure 12, the parameters is (*f_x_* = *f_y_* = *f_z_* = *f_φ_* = *f_ψ_* = *f_θ_* = 4), the fitness of the surveillance grows to 0.9908 while the number of the surveillance cameras is decreased to 16. From the experiments, we know that we can get more accurate results when we use a bigger sample space. In Figure 11, the sample space is 4,096 which is too big for classical Binary Integer Linear programming optimizations.

In the utility function, we set a regulization item which is a penalty for the camera number. We do an experiment to determine the effect of the *λ* parameter. In the next experiment we choose the task parameter as follows: surveillance area is 50 m × 50 m and the sample frequency is *f_x_* = *f_y_* = *f_z_* = *f_φ_* = *f_ψ_* = *f_θ_* = 4.

**Figure 13.**
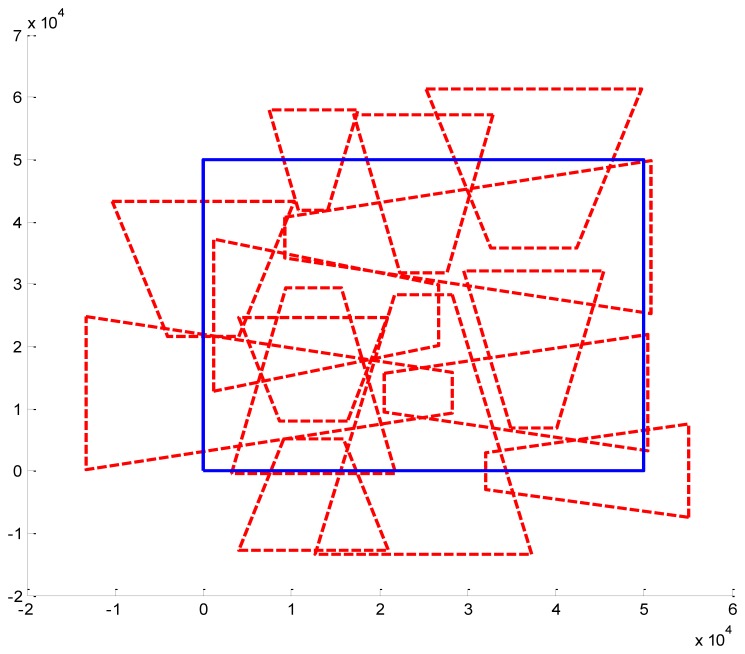
Optimal placement of cameras (14 cameras). The ssurveillance area is 50 m × 50 m, *f_x_* = 4, *f_y_* = 4, *f_z_* = 4, *f_φ_*=4, *f_θ_* = 4, *f_ψ_* = 4, *λ* = 2.

**Figure 14.**
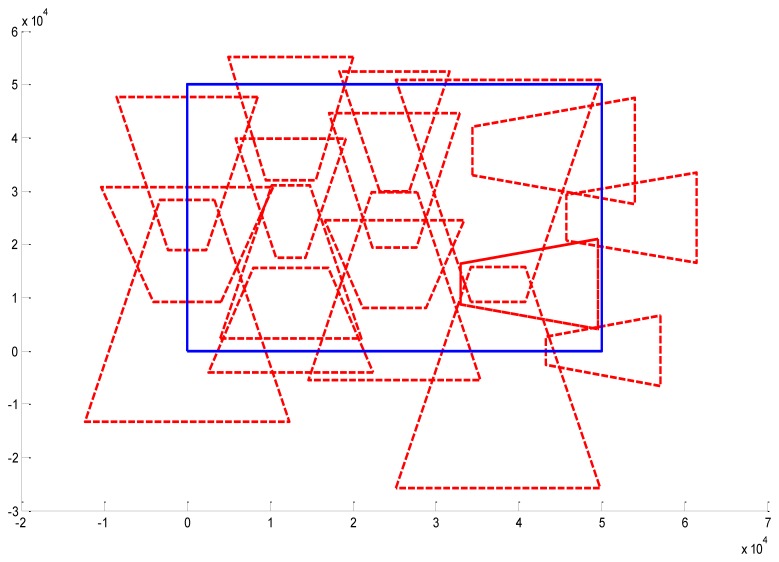
Optimal placement of cameras (18 cameras). The surveillance area is 50 m × 50 m, *f_x_* = 4, *f_y_* = 4, *f_z_* = 4, *f_φ_*=4, *f_θ_* = 4, *f_ψ_* = 4, *λ* = 0.1.

**Figure 15.**
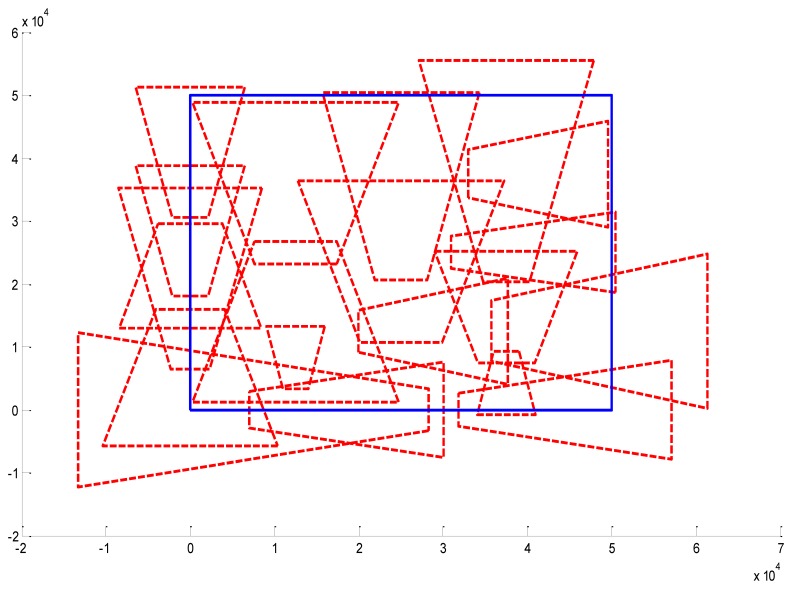
Optimal placement of cameras (20 cameras). The surveillance area is 50 m × 50 m, *f_x_* = 4, *f_y_* = 4, *f_z_* = 4, *f_φ_*=4, *f_θ_* = 4, *f_ψ_* = 4, *λ* = 0.01.

In Figure 13, the regulization parameter *λ* = 2. In Figure 14, the regulization parameter *λ* = 0.1. In Figure 15, the regulization parameter *λ* = 0.01. We can see that the regulization parameter takes effects to control the number of the cameras.

**Figure 16.**
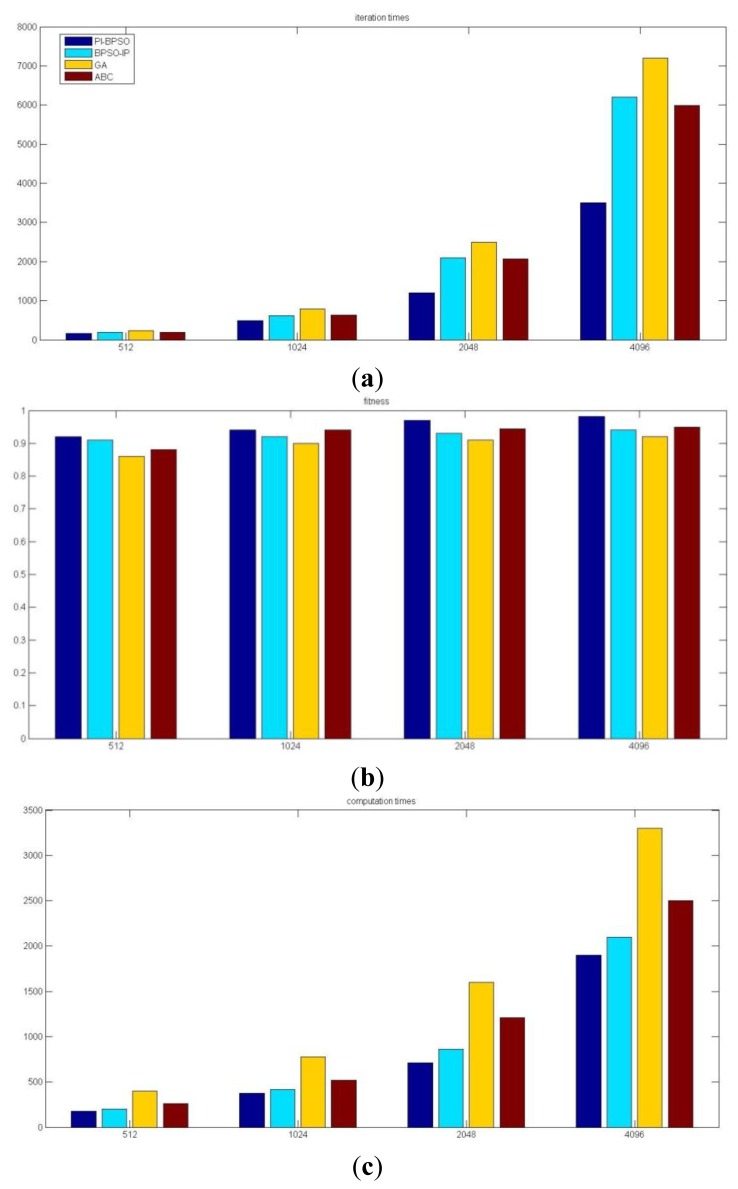
The comparison result of different optimization method. (**a**) The iteration times comparison result; (**b**) The fitness comparison result; (**c**) The computation time comparison result.

We do an experiment to compare PI-BPSO with BPSO-IP, GA and ABC from three perspectives: iteration times, fitness and computation time. We repeat the experiment 50 times and the value of the iteration times, fitness and computation time are the average values of the 50 tests. Because the codes of the other methods are all unlnown, the experimental results may not be same as the original author stated. We show the result in Figure 16.

**Figure 17.**
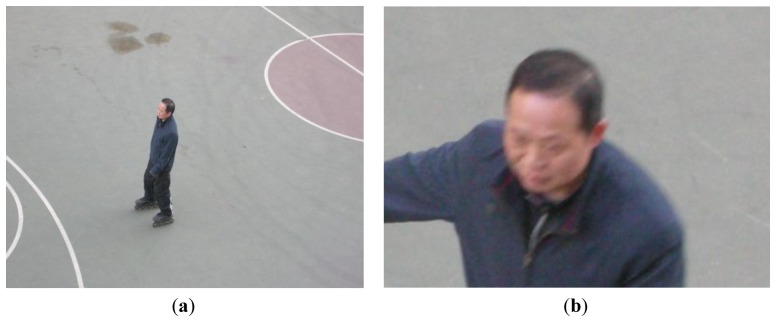
Some images from the real surveillence video to validate the effectiveness of the algorithm. (**a**) The video is in focus; (**b**) The resolution constraints are satisfied and the person can be identified in the video.

We do an experiment with real cameras in a hall building with the simulation result that we have obtained from the simulation. The surveillance area is a basketball court and a man is roller skating. We determine the position and pose of the surveillence cameras using the PI-BPSO algorithm. The experimental result shows the effect of the algorithm is that the video is in focus and the people in the video can be identified easily. We show the result in Figure 17.

## 6. Conclusions

In this paper, we discuss the automatic camera network placement problem which is solved by an evolution-like method, PI-BPSO. The different simulation results show the effectiveness of the proposed algorithm. The algorithm is guaranteed to get a global optimum with high probability due to two reasons:

(1) The initialization and the update process are both determined randomly which assures that we will now land in the local minimum with high probability;
(2) The introduction of the regulation item eases the optimization process and allows us to optimize the number of the cameras and the configuration of the cameras at the same time.

In the future, we will continue our study on the camera network placement problem from several directions, such as a more accurate initialization or integration of the PSO and other optimization methods to speed up the convergence.
